# Supplementary material for: Genomic analysis of Strongyloides stercoralis and Strongyloides fuelleborni in Bangladesh
Source: PLoS Negl Trop Dis. 2024 Sep 3;18(9):e0012440. doi: 10.1371/journal.pntd.0012440 (PMC11407627; doi:10.1371/journal.pntd.0012440)

Supplementary File 3 to

Genomic analysis of *Strongyloides stercoralis* and *Strongyloides fuelleborni* in Bangladesh

Veroni de Ree, Tilak Nath, Priyanka Barua, Dorothee Harbecke, Dongmin Lee, Christian Rödelsperger and Adrian Streit

Worms belonging to the "dog only" type show high apparent heterozygosity that is likely caused in part by structural variations, rather than true heterozygosity

When we attempted to include the "dog only" type worms (including the one isolated from a human host) in the heterozygosity analysis we noticed that they showed very high apparent heterozygosity. Strikingly, this was also the case for heterozygosity on the X chromosome in the male worm. To determine if this was an anomaly of our sample from Bangladesh we subjected the sequences of the five whole genome sequenced "dog only" type worms from [1] to the same analysis (Figure 8 in the body of the publication and Fig. Suppl_File_3_1). Except for the worm L6, these sequences showed even higher apparent heterozygosity, including on the X chromosome (all "dog only" type whole genome sequenced individuals by [1] were males). Notice that with respect to the mitochondrial and the nuclear genomes, L6 was the only whole genome sequenced worm in the study of [1] that belongs to a separate sub-cluster of the "dog-only" cluster, of which it is not clear, if it is more closely related to the other "dog only" cluster (to which the other four worms and the worms isolated in this study belong) or to the "human and dog" cluster (compare the positions of L6 in Figures 4-6 in the main body of the paper and Suppl. Figures 1, 2). For the rest of this Suppl. File 3 "dog only" always means "dog only" except for L6. L6 is not included in this analysis.

First, we asked if some of the *S. stercoralis* in Asia, in particular the "dog only" type might not employ XX/XO sex determination as it is the case in the USA derived *S. stercoralis* reference isolate [2]. We therefore performed read

coverage analysis and compared "dog only" cluster males, which have the high apparent heterozygosity on the autosomes and the X chromosome, with males and females from the reference isolate (which have low heterozygosity) and from "human and dog" cluster sequences from China, which have high heterozygosity but do not show the unexpected apparent heterozygosity on the X chromosome (Fig. Suppl_File_3_2). The proportional X- chromosome coverage in all males was comparable and clearly less than in females, suggesting that all these males do have only one X chromosome. Notice that free-living adults contain highly polyploid nuclei in the distal part of their gonads, in which the X chromosome is less amplified, compared with the autosomes in both sexes [3, 4]. Therefore, the overall coverage of the X chromosome in free-living adult females is less than 100% (compared with autosomes) but still more than 50% (in our cases between 70 and 80%) and less than the expected 50% in males [2, 4]. In infective larvae, all of which are female and do not have the polyploid nuclei yet, the coverage of the X is equal to the autosomes (Fig. Suppl_File_3_2E)

We then analysed the heterozygosity over the length of the chromosomes. Males did indeed show low heterozygosity over large portions of the X chromosome compared with females and with autosomes of both sexes (Fig. Suppl_File_3_3). However, there are apparent heterozygosity hot spots that dominated the analysis. These hotspots are also present in females. Also on autosomes, such heterozygosity hotspots are visible, although less frequent. We think these hotspots reflect duplications and X to autosome translocations present in the genome of the "dog only" type, compared with the *S. stercoralis* reference genome [2].

Overall, we think that the heterozygosity detected in the "dog only" worms is at least partially artificial due to the use of a too different reference genome that does not contain some duplications and X to autosome translocations present in these worms. Currently we cannot really tell what portion of the apparent heterozygosity is real and how much is artificial. The problem appears to be aggravated on the X chromosome because also females show higher heterozygosity on the X chromosome than on the autosomes. Also, in the reference genome, the assembly of the autosomes is better than the one of the X chromosome. Some of the low but detectable apparent heterozygosity seen on the X in males of the human derived *S. stercoralis* might also be caused artificially due to assembly issues.

Figure legends

Fig. Suppl_File_3_1: Apparent heterozygosity of "human and dog" type worms from this and previous studies [[5] Thailand, [6] Japan and Myanmar, [7] Iran, [8] China, [1] Cambodia and [2] USA] plus the "dog only" type worms from this study and from [1]. The X axis shows the heterozygosity on the autosomes, the Y axis the heterozygosity on the X chromosome. The diagonal indicates equal heterozygosity on the X chromosome and the autosomes. The labels only refer the circled worms in the same colour as the label. Notice the high heterozygosity on the X chromosome in males (squares) of the "dog only" type. In particular, compare the human derived worms with hight heterozygosity from China (green) with the dog derived samples. For a discussion of the one female in the male group from China see [8]. Notice that for the "dog only" worms the females

are above the diagonal while the males are on or below the diagonal, indicating inflated X chromosomal heterozygosity in females as well. For the details of the analysis see Materials and Methods in the main text of this publication.

Fig. Suppl_File_3_2: Read coverage of individual male and female worms. The reads from single worm Illumina sequencing were aligned with the reference genome [2] and for each position the coverage was determined. The x-axes show the coverage, the y-axes show the number of positions with the corresponding coverage for the autosomes (blue) and the X chromosome (red). the % indicate the relative coverage of the X chromosome compared with the autosomes. Notice that due to the uneven amplification of the genomic DNA in the highly polyploid nuclei of the distal gonad the X chromosome is underrepresented in free living adults of both sexes [2, 4]. (A,B) females and (C,D) males of the reference isolate (data from [2]. (E-H) worms from the study by [8] in China. (E) infective larva, which is female but has no polyploid germline cells. (F-H) males that have high heterozygosity on the autosomes but not on the X chromosome. The coverage of the X chromosome is comparable with the one in the reference males, confirming that these males have only one X chromosome. Notice that no free-living females were found by [8]. (I,K) dog only type worms from [1] that have high apparent heterozygosity on the autosomes and on the X chromosome (Fig. Suppl_File_3_1). The coverage of the X chromosome is comparable with the reference males suggesting these males have only one X chromosome. Notice the rather high number of low or completely not covered positions and the irregular shape of the graph towards the left of the graph. We think that this is caused by the rather

large sequence and genome structural differences between these sequences and the reference sequence. The sequencing depth in the Bangladesh study (this manuscript) was lower such that a reliable quantitative comparison between the sexes was not possible, but the coverage of the X in males was clearly lower than in females in this study as well.

Fig. Suppl_File_3_3: Distribution of heterozygous positions over the three largest X chromosomal contigs (A) and over the two largest autosomal scaffolds (B). The x-axes show the position along the contig/scaffold and the y-axes show the number of apparently heterozygous positions per 10 kb window. The black lines represent the running means. Three females and the one male from the "dog only clade" of this study are shown. Notice the very low number of heterozygous positions on the X chromosome in the male over most of the X chromosomal contigs and the apparent hotspots (arrows). The same hotspots are also visible in the females. Hotspots are also present on the autosomes, although less frequent (notice that the autosomal scaffolds are much longer than the X chromosomal ones.

References

1. Jaleta TG, Zhou S, Bemm FM, Schar F, Khieu V, Muth S, et al. Different but overlapping populations of *Strongyloides stercoralis* in dogs and humans-Dogs as a possible source for zoonotic strongyloidiasis. PLoS Negl Trop Dis. 2017;11(8):e0005752. doi: 10.1371/journal.pntd.0005752. PubMed PMID: 28793306; PubMed Central PMCID: PMCPMC5565190.

2. Hunt VL, Tsai IJ, Coghlan A, Reid AJ, Holroyd N, Foth BJ, et al. The genomic basis of parasitism in the *Strongyloides* clade of nematodes. Nat Genet. 2016;48(3):299-307. doi: 10.1038/ng.3495. PubMed PMID: 26829753.

3. Hammond MP, Robinson RD. Endoreplication in the ovary, testis, and intestine of Strongyloides stercoralis. J Parasitol. 1994;80(6):905-10. PubMed PMID: 7799163.

4. Kulkarni A, Holz A, Rodelsperger C, Harbecke D, Streit A. Differential chromatin amplification and chromosome complements in the germline of Strongyloididae (Nematoda). Chromosoma. 2016;125(1):125-36. doi: 10.1007/s00412-015-0532-y. PubMed PMID: 26205504.

5. Aupalee K, Wijit A, Singphai K, Rodelsperger C, Zhou S, Saeung A, et al. Genomic studies on *Strongyloides stercoralis* in northern and western Thailand. Parasites & vectors. 2020;13(1):250. Epub 2020/05/15. doi: 10.1186/s13071-020-04115-0. PubMed PMID: 32404172; PubMed Central PMCID: PMCPMC7222524.

6. Kikuchi T, Hino A, Tanaka T, Aung MP, Afrin T, Nagayasu E, et al. Genome-Wide Analyses of Individual *Strongyloides stercoralis* (Nematoda: Rhabditoidea) Provide Insights into Population Structure and Reproductive Life Cycles. PLoS Negl Trop Dis. 2016;10(12):e0005253. doi: 10.1371/journal.pntd.0005253. PubMed PMID: 28033376; PubMed Central PMCID: PMC5226825.

7. Beiromvand M, Ashiri A, de Ree V, Harbecke D, Rodelsperger C, Streit A, et al. *Strongyloides stercoralis* genotyping in a human population in southwestern Iran. Parasites & vectors. 2024;17(1):21. Epub 20240116. doi: 10.1186/s13071-023-06103-6. PubMed PMID: 38229164; PubMed Central PMCID: PMCPMC10792921.

8. Zhou S, Fu X, Pei P, Kucka M, Liu J, Tang L, et al. Characterization of a non-sexual population of *Strongyloides stercoralis* with hybrid 18S rDNA haplotypes in Guangxi, Southern China. PLoS Negl Trop Dis. 2019;13(5):e0007396. Epub 2019/05/07. doi: 10.1371/journal.pntd.0007396. PubMed PMID: 31059500.

Fig_Suppl_File_3_1

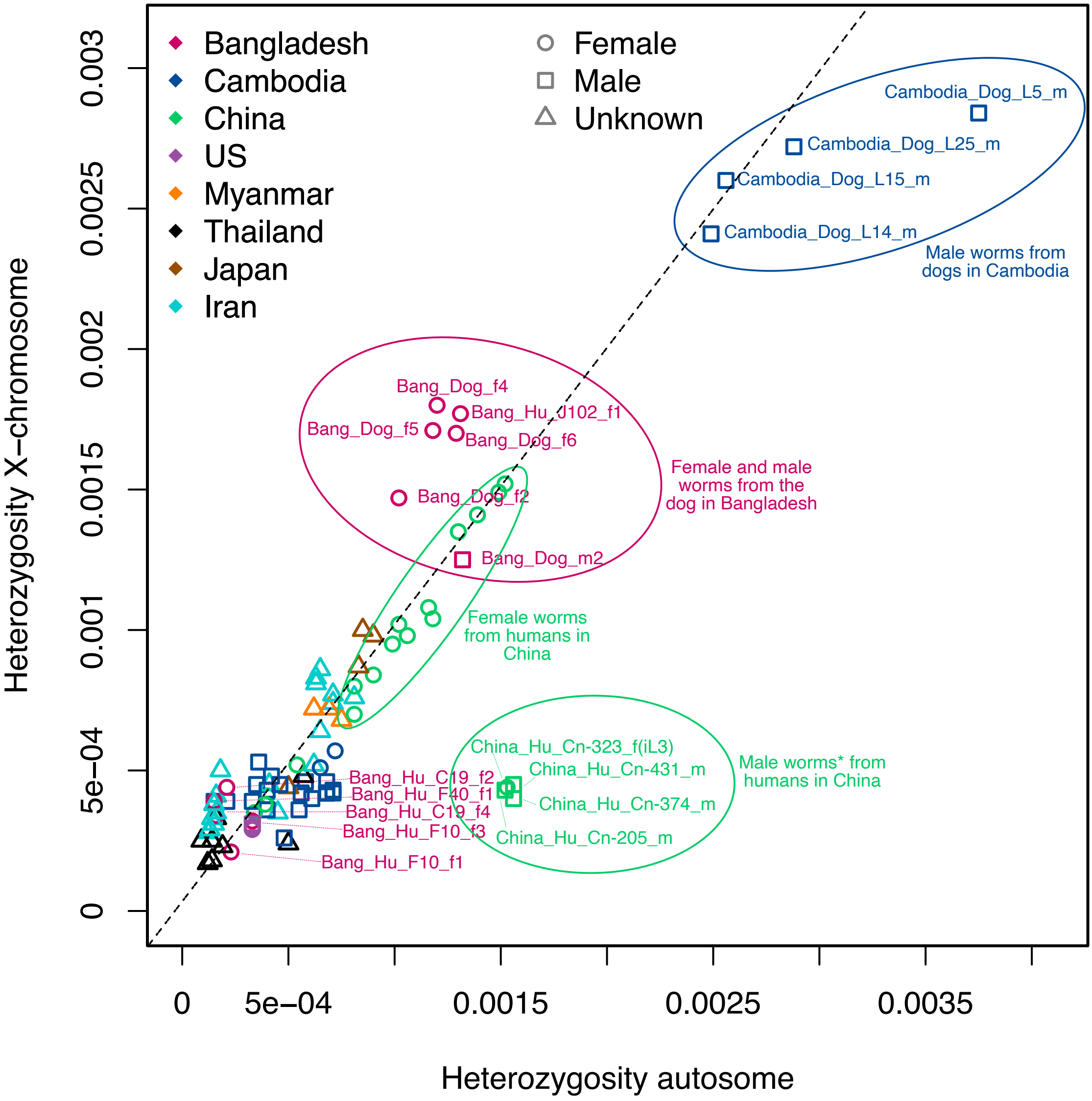

Fig_Suppl_File_3_2

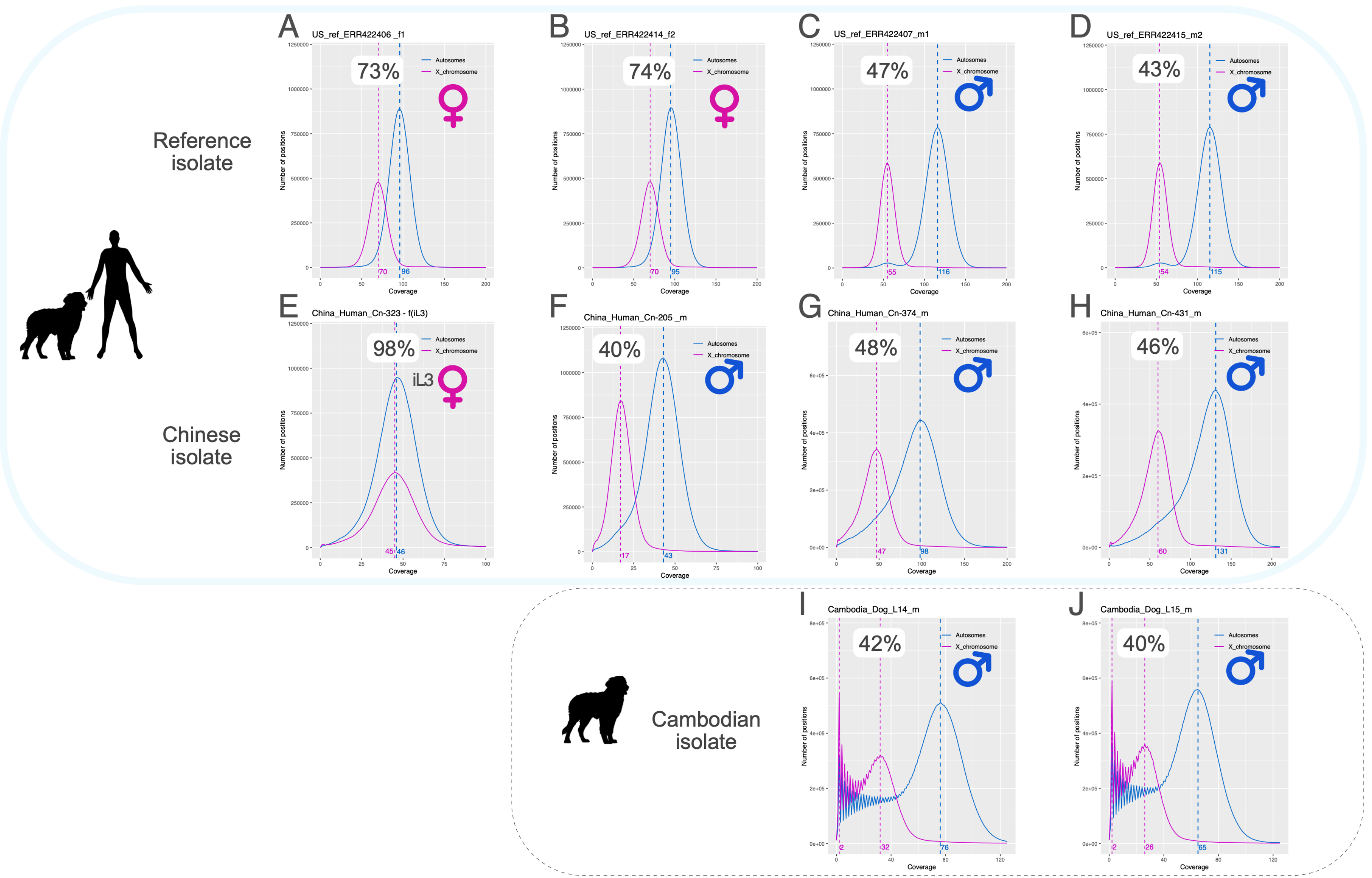

Fig_Suppl_file_3 3

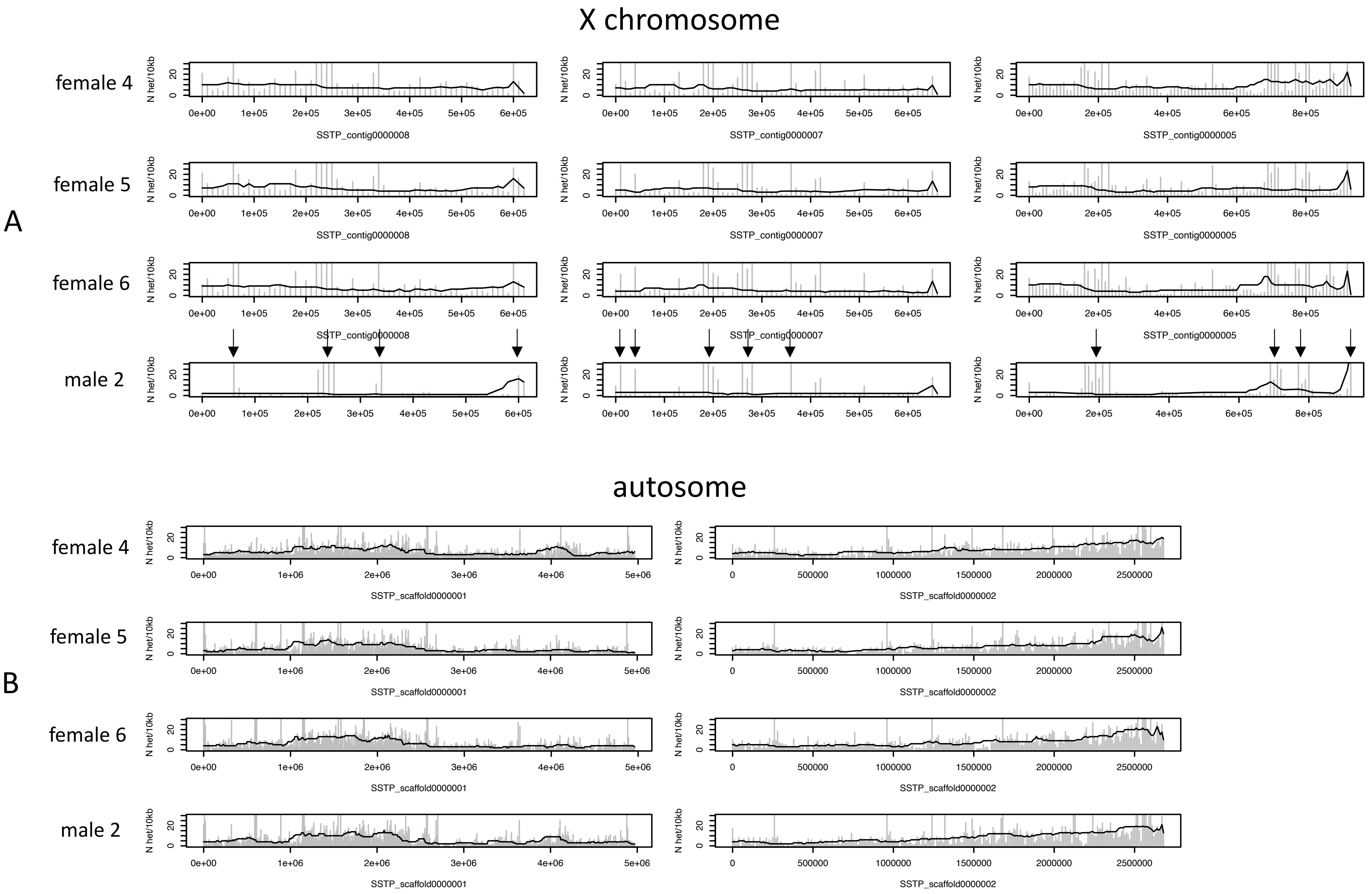

Supplement: S3 File — (PDF) [file pntd.0012440.s003.pdf]
