# Supplementary figures and images for: Genomic analysis of Strongyloides stercoralis and Strongyloides fuelleborni in Bangladesh
Source: PLoS Negl Trop Dis. 2024 Sep 3;18(9):e0012440. doi: 10.1371/journal.pntd.0012440 (PMC11407627; doi:10.1371/journal.pntd.0012440)

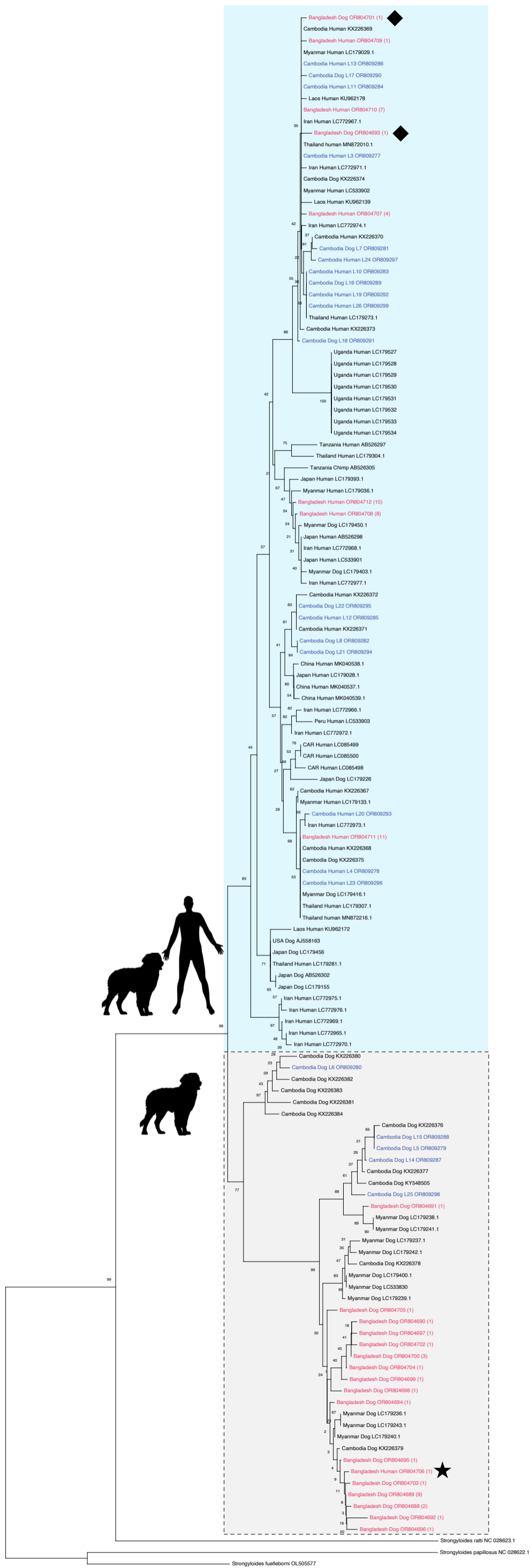

0.050

Supplement: S1 Fig — Diamonds label the two worms that showed "human and dog" type mitochondrial but "dog only" type nuclear sequences. The asterisk labels the worm in the "dog only" cluster isolated from a human host. Sequences found in this study are in red, the number of worms this sequence was found in is in (). Samples in blue are worms from [20] for which full genome short read sequences are available. For every sample the country of origin (CAR = Central African Republic, USA = United States of America), the host and the GenBank accession number are given. For worms from Cambodia also the worm individual is given after the host to facilitate cross reference with [20]. Samples from Cambodia are from [20], samples from Laos are from [39], samples from Myanmar, Thailand and Uganda are from [26], samples from Iran are from [25], sample from the USA is from [40], samples from Tanzania, and Japan are from [17], samples from CAR are from [41] and samples from China are from [24]. (PDF) [file pntd.0012440.s005.pdf]

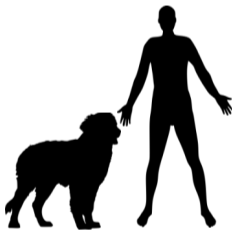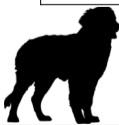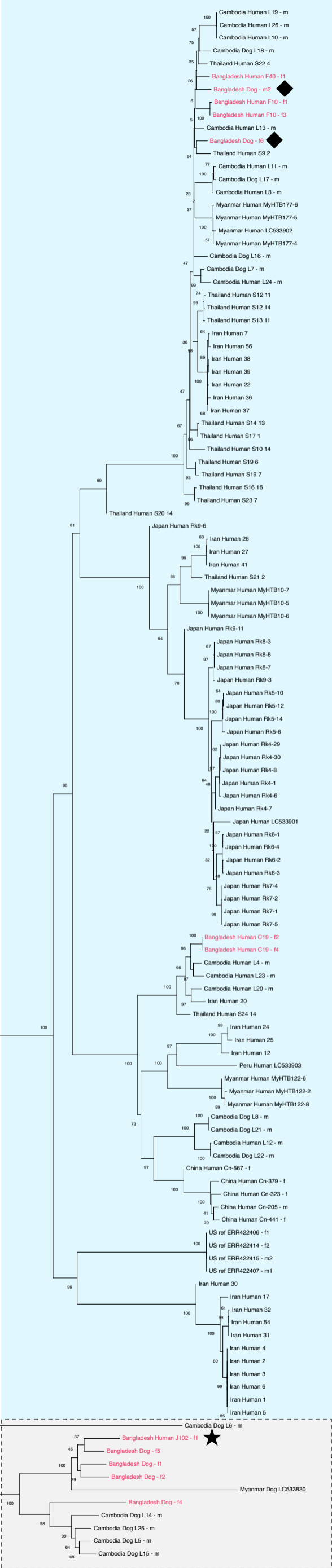

Supplement: S2 Fig — Diamonds label the two worms that showed "human and dog" type mitochondrial but "dog only" type nuclear sequences. The asterisk labels the worm in the "dog only" cluster isolated from a human host. The sequences from this study are in red. The other sequences were extracted from the whole genome sequencing read data of the following references: Cambodia [20], Thailand [22], Iran [25], Myanmar and Japan [23], USA [44] and China [24]. A FASTA file with all sequences used is provided as S2 File. (PDF) [file pntd.0012440.s006.pdf]

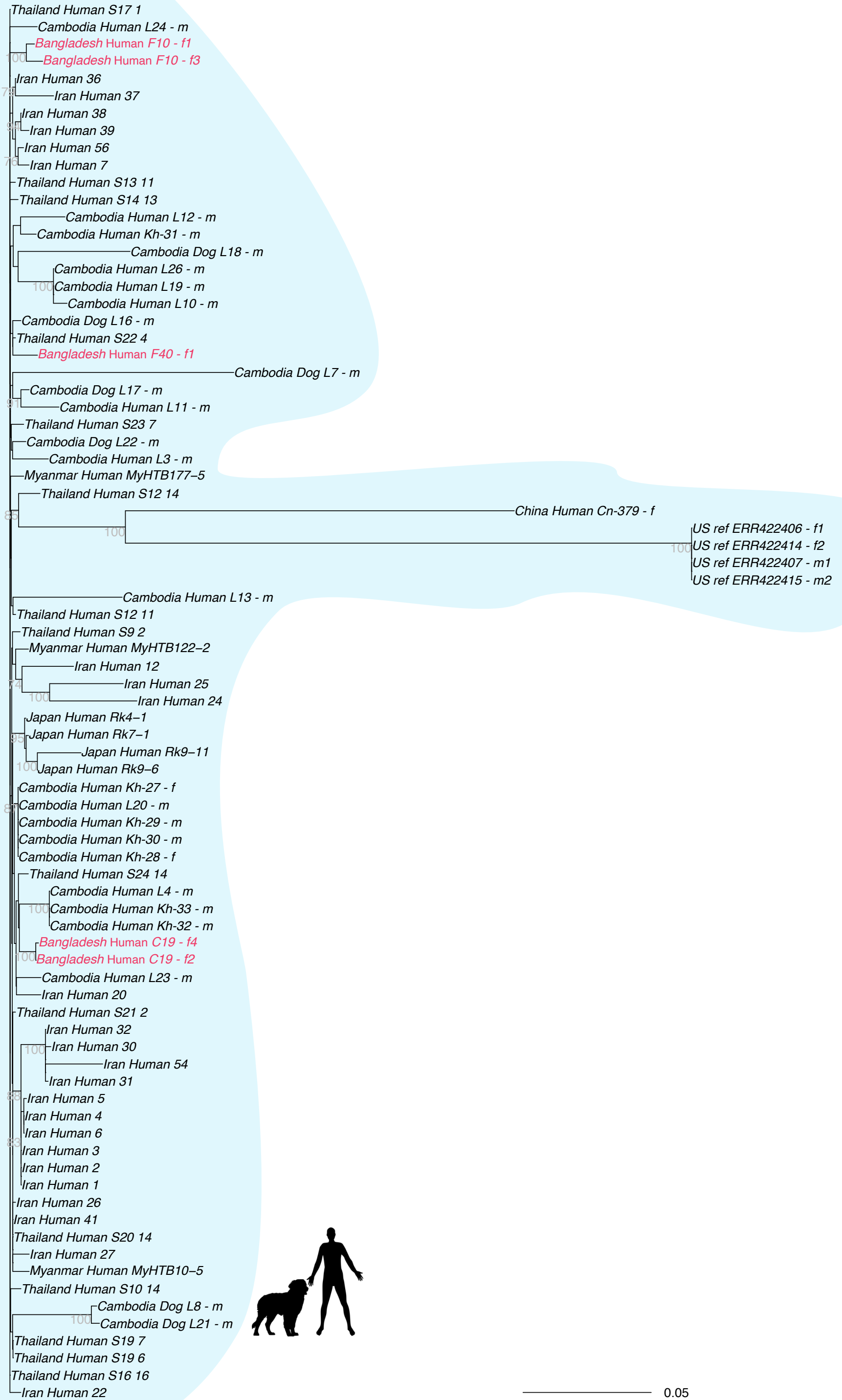

Supplement: S3 Fig — The sequences from this study are in red. The other sequences were extracted from the whole genome sequencing read data of the following references: Cambodia [20], Thailand [22], Iran [25], Myanmar and Japan [23], USA [44] and China [24]. (PDF) [file pntd.0012440.s007.pdf]

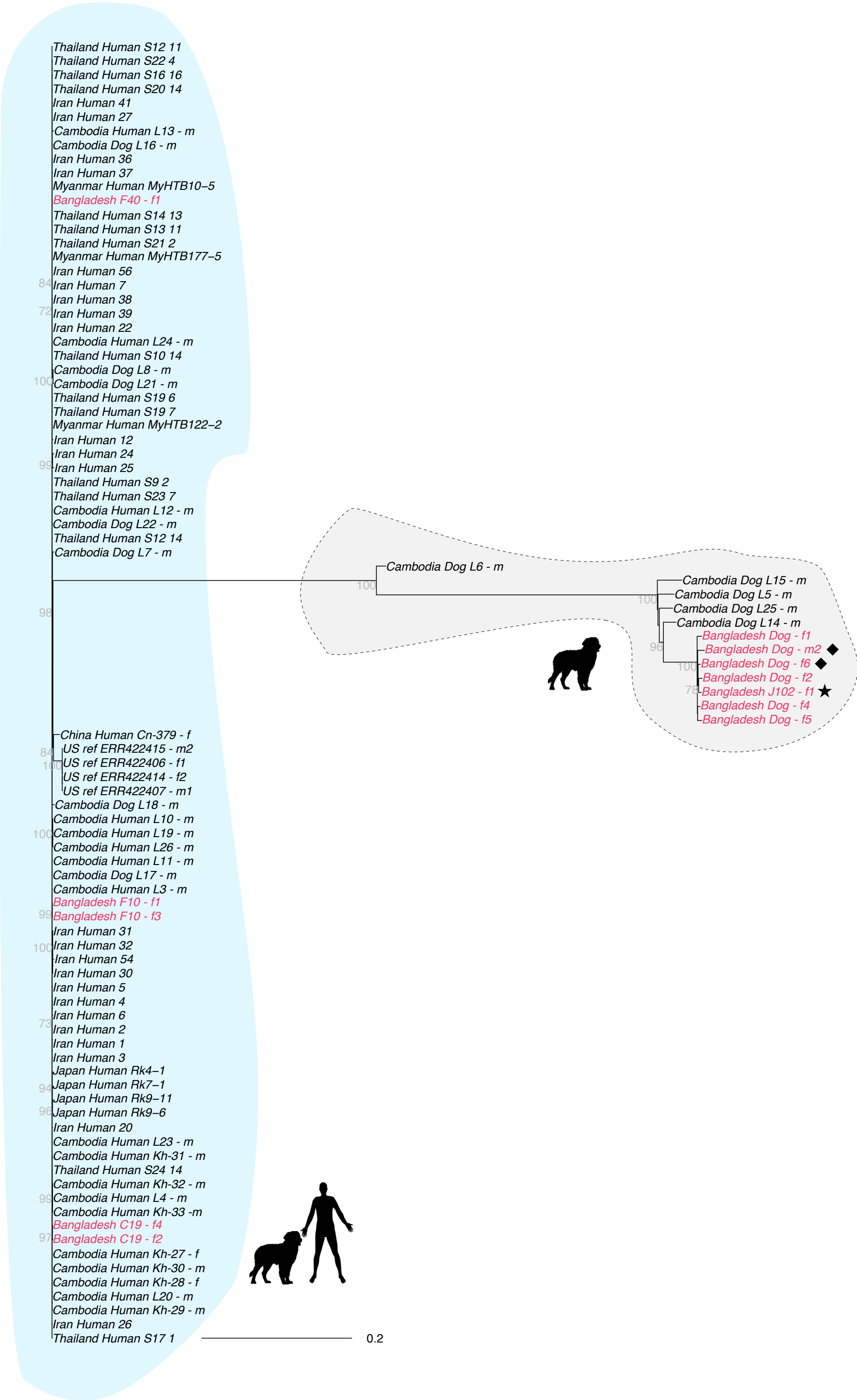

Supplement: S4 Fig — Diamonds label the two worms that showed "human and dog" type mitochondrial but "dog only" type nuclear sequences. The asterisk labels the worm in the "dog only" cluster isolated from a human host. The sequences from this study are in red. The other sequences were extracted from the whole genome sequencing read data of the following references: Cambodia [20], Thailand [22], Iran [25], Myanmar and Japan [23], USA [44] and China [24]. (PDF) [file pntd.0012440.s008.pdf]
